# Supplementary material for: Population genomics of the pathogenic yeast Candida tropicalis identifies hybrid isolates in environmental samples
Source: PLoS Pathog. 2021 Mar 31;17(3):e1009138. doi: 10.1371/journal.ppat.1009138 (PMC8041210; doi:10.1371/journal.ppat.1009138)
Supplement: S1 Text — (DOCX) [file ppat.1009138.s001.docx]

**S1 Text. LOH analysis using updated chromosome-level reference genome assembly**

Loss of heterozygosity (LOH) was analysed using the *Candida tropicalis* reference genome, Assembly B, as described in Materials and Methods. This genome sequence was based on the original *C. tropicalis* reference genome published by Butler et al. (2009), which consisted of 23 scaffolds. We improved this reference sequence by joining several of these scaffolds based on synteny with other CTG-Ser1 clade species to produce Assembly B. This sequence was subsequently further improved by Guin et al. (2020), generating a complete chromosome-level assembly with seven chromosomes. We re-analysed loss of heterozygosity in the 77 *C. tropicalis* isolates using the updated reference genome, as described in Materials and Methods.

The LOH results were almost identical using the updated reference genome, as compared to Assembly B. We again observed a pattern of heterozygous regions alternating with homozygous, or LOH, regions in all *C. tropicalis* isolates (Fig. S5A). The average proportion of the non-hybrid (AA) genomes classified as heterozygous or LOH remains the same, at 4% and 95%, respectively (Table S6). The mean length of heterozygous and LOH blocks in AA isolates remains almost the same at 209 bp and 1.9 kb, respectively. In the homozygous isolate, *C. tropicalis* ct20, we observe the same pattern of extensive LOH when compared to the alternative reference genome, with 0.37% of the genome classified as heterozygous and a mean heterozygous block length of 217 bp. As in our original analysis, >99% of this genome consists of regions that have undergone LOH.

The LOH observed in the hybrid genomes was also similar. On average, 69% of the six hybrid (AB/AC) genomes is classified as heterozygous and 30% is classified as LOH, matching our initial analysis. The six hybrid genomes have an average of 13,152 LOH blocks per isolate, covering between 25.6 and 42.6% of the genome. The mean length of LOH regions in these isolates is 334 bp, with a maximum length of 112 kb (Fig. S5B).

Our re-analysis of LOH and heterozygous regions in the genomes of the hybrids suggests that some of the hybrids share a common origin, consistent with our initial observations. When we re-analysed shared LOH regions using the alternative reference genome, only 1.6% of LOH blocks (equating to 739 LOH blocks) is conserved among all six isolates. This result is almost identical to our initial observations with Assembly B. We observed the same degree of LOH conservation in the four AB hybrids (~17%) and in the AC isolates (~55%) when analysed using the alternative reference genome, as compared to Assembly B.

We also observed partial conservation of the large region of LOH observed by Guin et al. (2020) on chromosome R (scaffold 4 of the original reference genome) of the hybrid genomes, in agreement with our initial analysis using Assembly B. The region of LOH identified by Guin et al. (2020) covered the left arm of chromosome R, from the telomere to the centromere, including the rDNA locus (approximately 800 kb). In both LOH analyses we observed long tracts of LOH at this region, up to and including the rDNA locus (approximately 400 kb) in four of the hybrid isolates (*C. tropicalis* ct25, ct42, ct77 and ct78). We observed a smaller region of LOH in two of the AB hybrids (*C. tropicalis* ct75 and ct76) covering only the rDNA locus (approximately 30 kb).

Conservation of heterozygous regions was also examined using both Assembly B and the alternative reference genome. Both analyses gave almost identical results. Using the alternative reference genome, shared heterozygous regions in all six hybrid isolates cover 6.1 Mb, with 45% of the 511,856 heterozygous sites in these regions sharing a common allele in all six isolates. This is in agreement with our initial analysis using Assembly B, and again indicates that the six hybrid isolates did not originate from the same parental strains. We observe a much higher degree of conservation of variants in shared heterozygous regions among the AB isolates; 94% of 442,231 heterozygous variants in 7.0 Mb are present in all AB isolates. Similarly, the two AC hybrids share 98% of 651,332 variants across 10 Mb. The proportion of shared heterozygous variants in shared heterozygous regions in the alternative reference genome (i.e. 94 and 98%) is the same as in Assembly B. This is in agreement with our initial analysis that the four AB isolates share a common origin, and that the two AC isolates share a separate, common origin.
