# Supplementary figures and images for: Population genomics of the pathogenic yeast Candida tropicalis identifies hybrid isolates in environmental samples
Source: PLoS Pathog. 2021 Mar 31;17(3):e1009138. doi: 10.1371/journal.ppat.1009138 (PMC8041210; doi:10.1371/journal.ppat.1009138)

**A**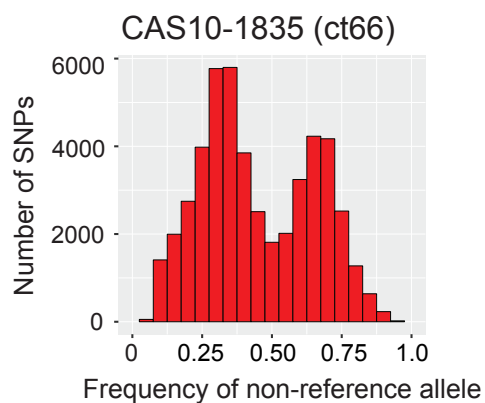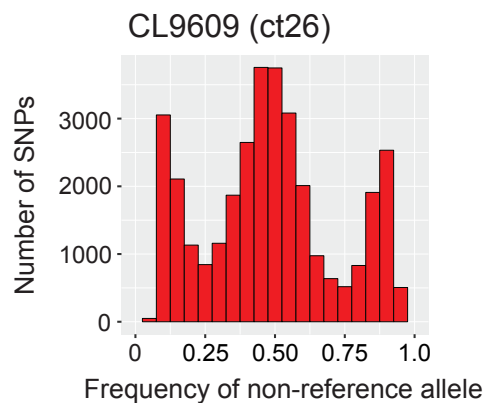**B**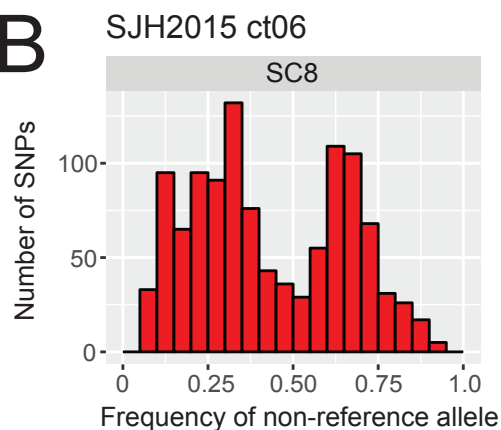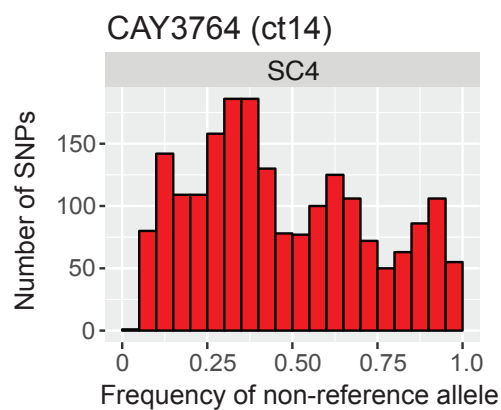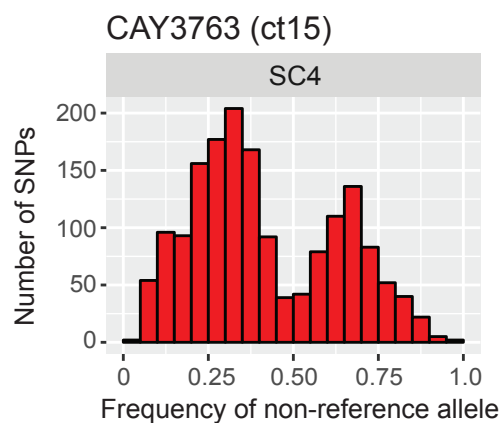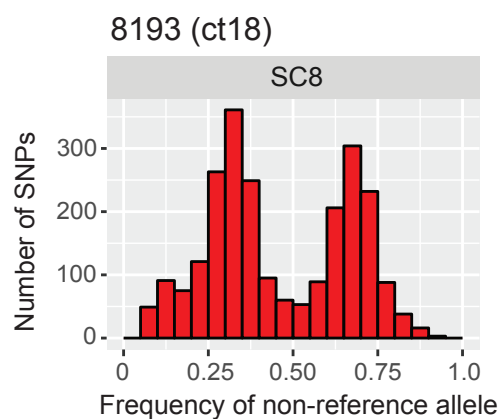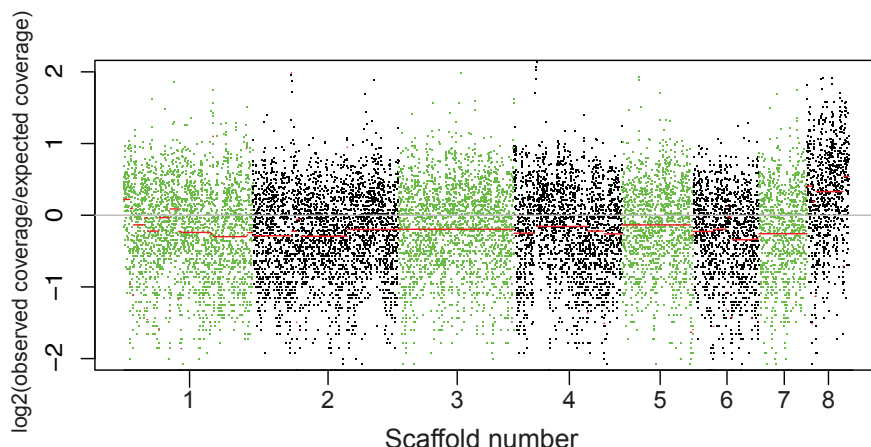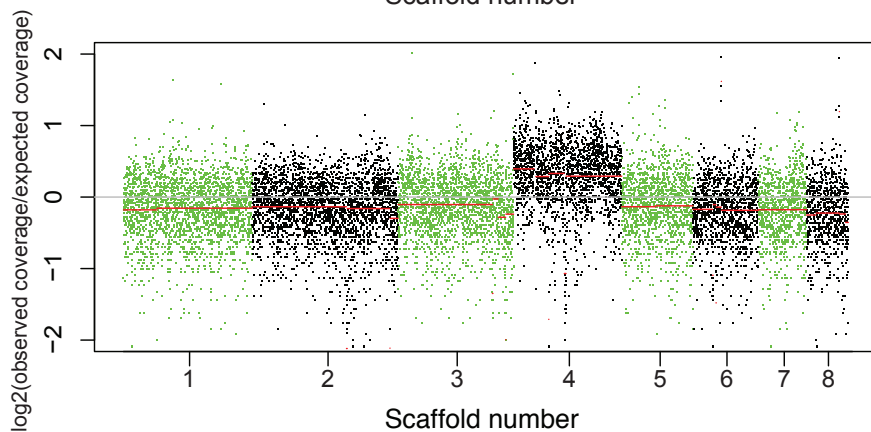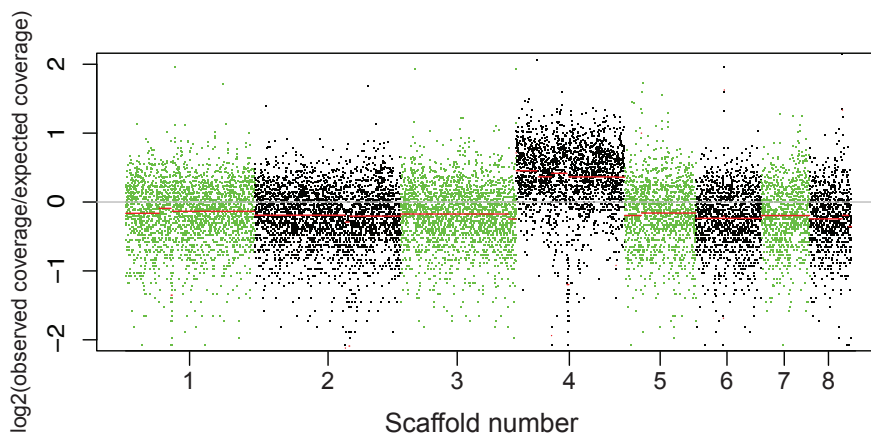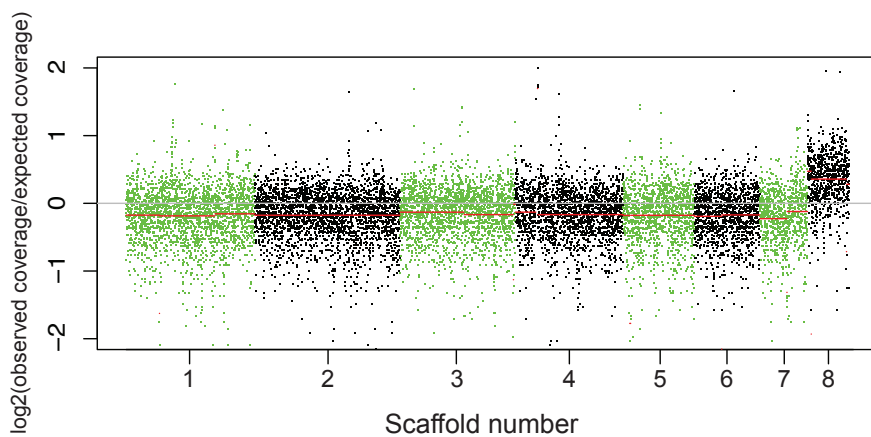

Supplement: S1 Fig — (A) Polyploidy of C. tropicalis isolates. The frequency of the non-reference allele for all heterozygous biallelic SNPs across all scaffolds is shown for each of the isolates, with frequency on the Y-axis and alternate (non-reference) allele frequency on the X-axis. For each SNP, allele frequency was calculated as the depth of the alternate allele divided by the total depth at the variant site. Triploidy of C. tropicalis ct66 is indicated by peaks of allele frequency at 0.33 and 0.66. Octaploidy of C. tropicalis ct26 is indicated by peaks of allele frequency at approximately 0.5, 0.12 and 0.87. Allele frequencies of approximately 0.125 and 0.875 imply that seven chromosomes carry one allele, and one chromosome carries a second allele. In this isolate, we also observe a peak at 0.5, implying that in some cases, four chromosomes carry one allele and four chromosomes carry a second allele. This multimodal distribution (i.e. peaks at 0.125, 0.50 and 0.875) is likely to be the result of loss of heterozygosity (LOH) affecting portions of some scaffolds, leading to a pattern wherein some variant sites have a 4:4 ratio of reference:non-reference allele frequency and some have a 7:1 ratio. (B) Aneuploidy of C. tropicalis isolates. Single chromosome aneuploidies were identified in four isolates; C. tropicalis ct06, a clinical isolate from Dublin, Ireland, C. tropicalis ct14 and ct15, both engineered strains from the USA [41], and C. tropicalis ct18, a clinical isolate from Madrid, Spain. Aneuploidies were identified by patterns in the distribution of allele frequency in heterozygous biallelic SNPs (shown as red histograms for the relevant scaffold, with frequency on the Y-axis and alternative allele frequency on the X-axis). Allele frequency was calculated as the depth of coverage of the alternate (non-reference) allele divided by the total depth at the variant site. Aneuploidies were confirmed by elevated coverage at the relevant locus (shown as dot plots, with green and bla [file ppat.1009138.s002.pdf]

A

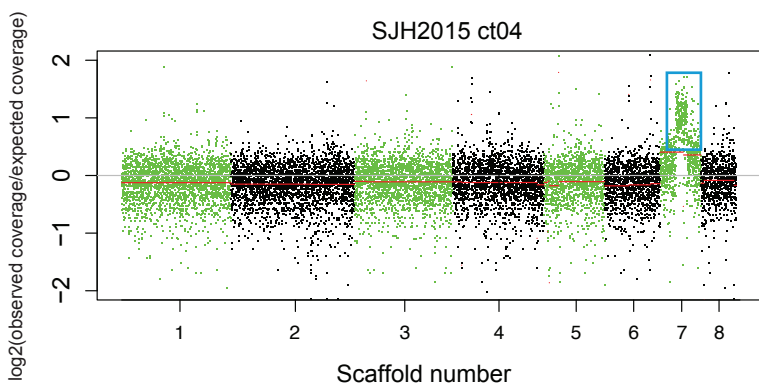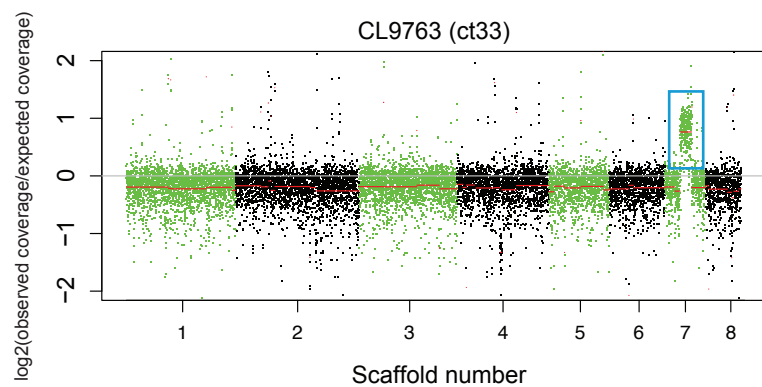

B

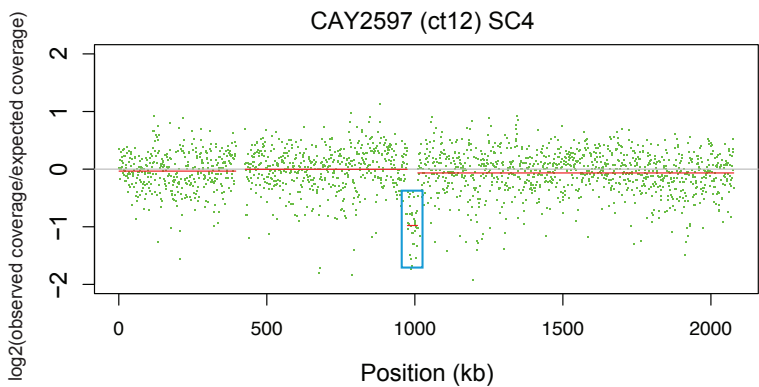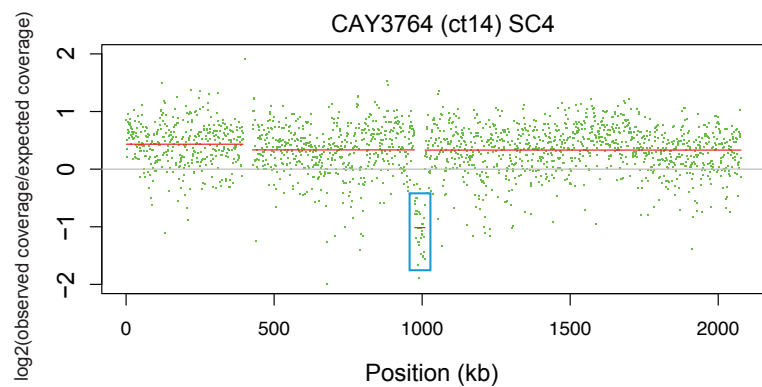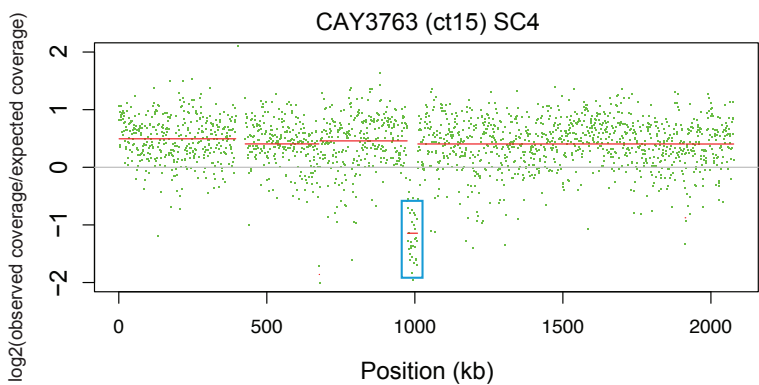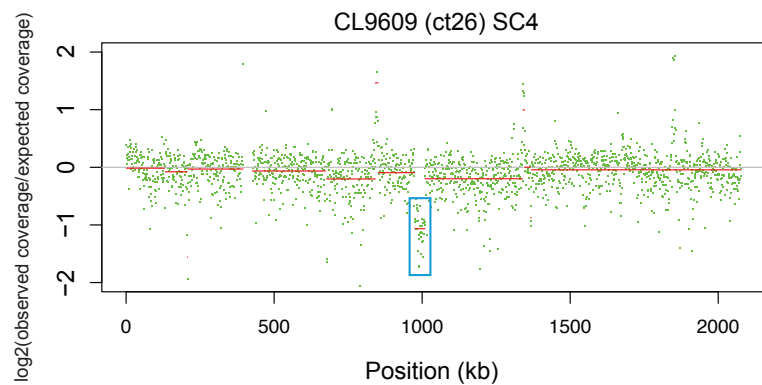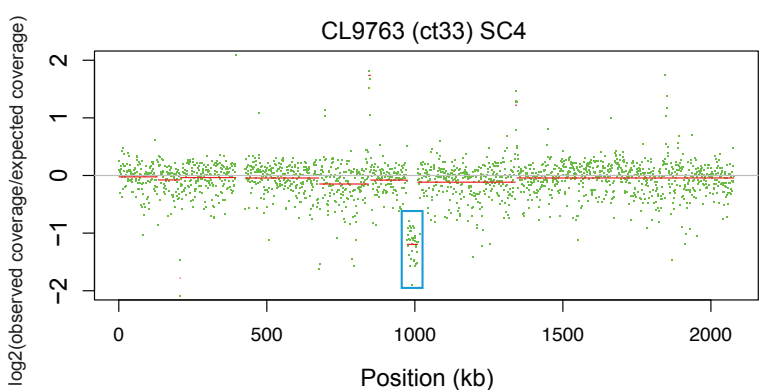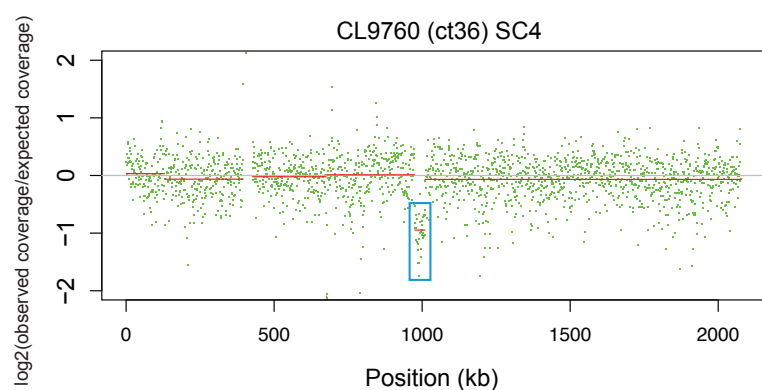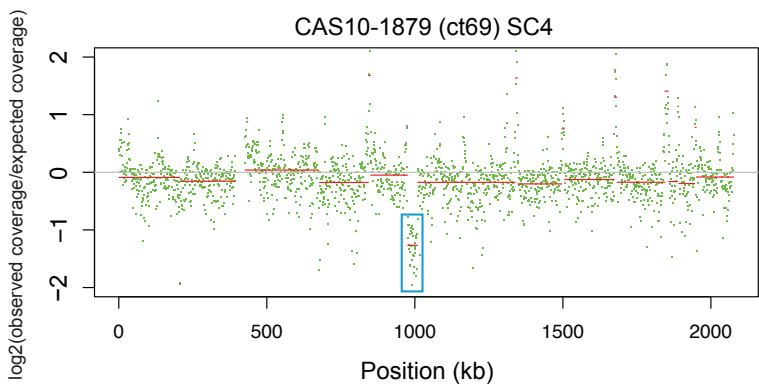

Supplement: S2 Fig — (A) CNV in isolates C. tropicalis ct04 and C. tropicalis ct33. CNVs were visualized as elevated coverage at the relevant locus (shown as dot plots, with green and black representing alternating scaffolds). Scaffold number is shown on the X-axis and the log2(observed coverage/expected coverage) is shown on the Y-axis (where “expected coverage” is the average genome-wide coverage for that isolate). Scaffolds are listed in decreasing order of size; the eight largest scaffolds are shown. A duplication of a region of approximately 253 kb on scaffold 7 is observed in two isolates; C. tropicalis ct04, a clinical isolate from Dublin, Ireland, and C. tropicalis ct33, a clinical isolate from Madrid, Spain. This CNV (highlighted with a blue box) spans the region from approximately 350 kb to 603 kb. A score of 1 at this region indicates a doubling in coverage, i.e. a total copy number of four. (B) CNV in isolates ct12, ct14, ct15, ct26, ct33, ct36 and ct69. CNVs were visualized as elevated coverage at the relevant locus (shown as dot plots). Position on the chromosome (kb) is shown on the X-axis and the log2(observed coverage/expected coverage) is shown on the Y-axis (where “expected coverage” is the average genome-wide coverage for that isolate). Scaffold 4 only is shown. A small CNV (~35 kb) is visible at the 1 Mb point of scaffold 4 in seven isolates, C. tropicalis ct12 (a clinical isolate from Colombia), ct14, ct15 (both engineered isolates from the USA), ct26, ct33, ct36 (three clinical isolates from Madrid, Spain) and ct69 (a clinical isolate from the USA). This CNV (highlighted with a blue box) spans the region from approximately 974 kb to 1.009 Mb on scaffold 4. A score of -1 at this region indicates a relative coverage level of 0.5. (PDF) [file ppat.1009138.s003.pdf]

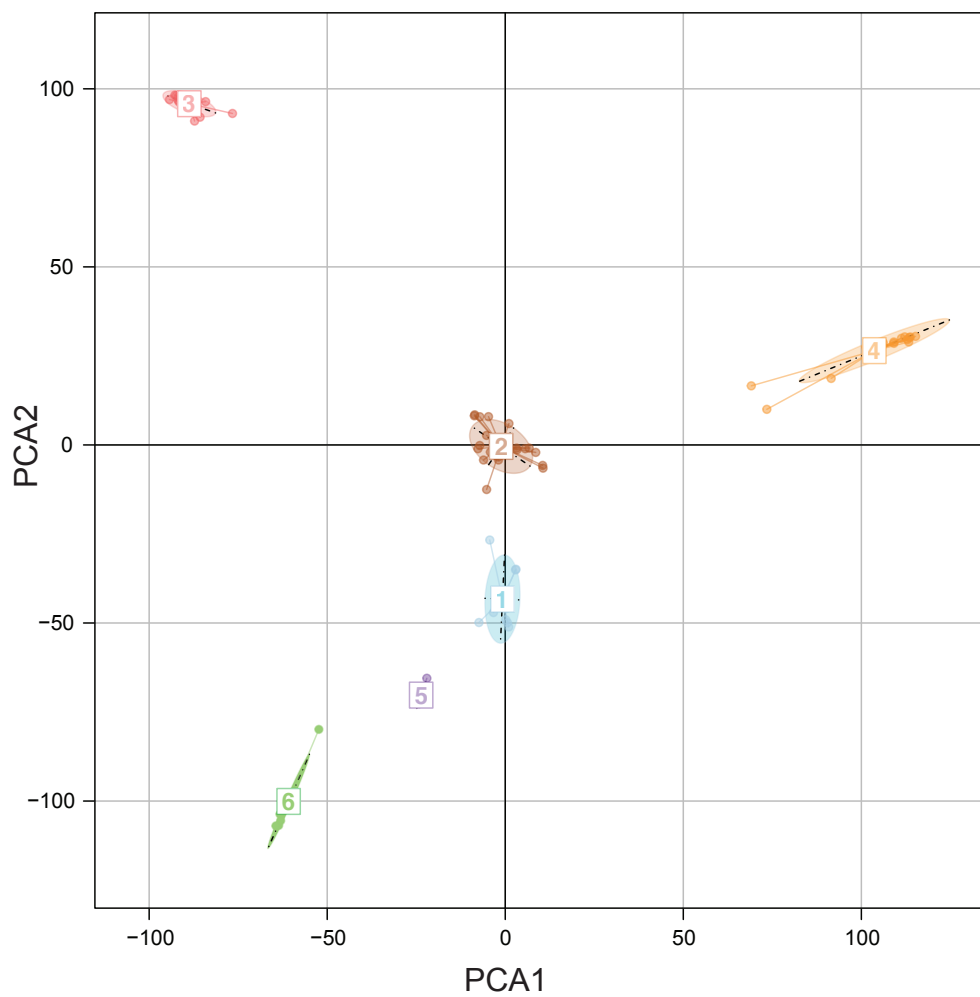

Supplement: S3 Fig — Principal component analysis (PCA) of Cluster A isolates (Fig 1B) was performed using the ade4 package in R [95] (S4 Table). Principal components 1 and 2 are represented on the X- and Y-axes respectively. Six clusters were identified using Ward’s method. Clusters one, three, four, five and six are the same as groupings as Fig 1C, except that C. tropicalis ct09 is included in Cluster 4 in the PCA analysis only, and C. tropicalis ct38 is included in Cluster 1 in the PCA analysis only. Cluster 2 is not clearly separated in the SNP phylogeny. (PDF) [file ppat.1009138.s004.pdf]

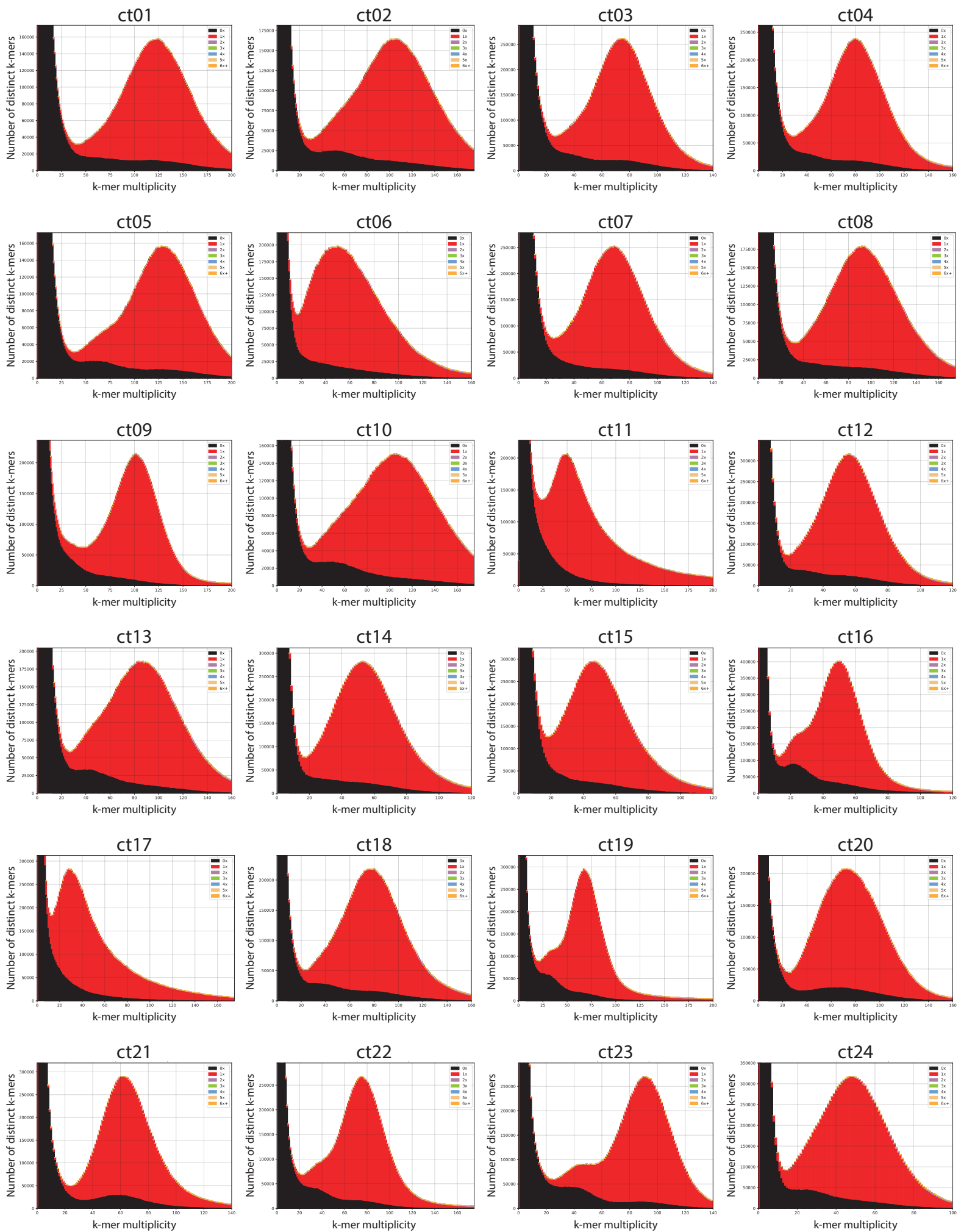

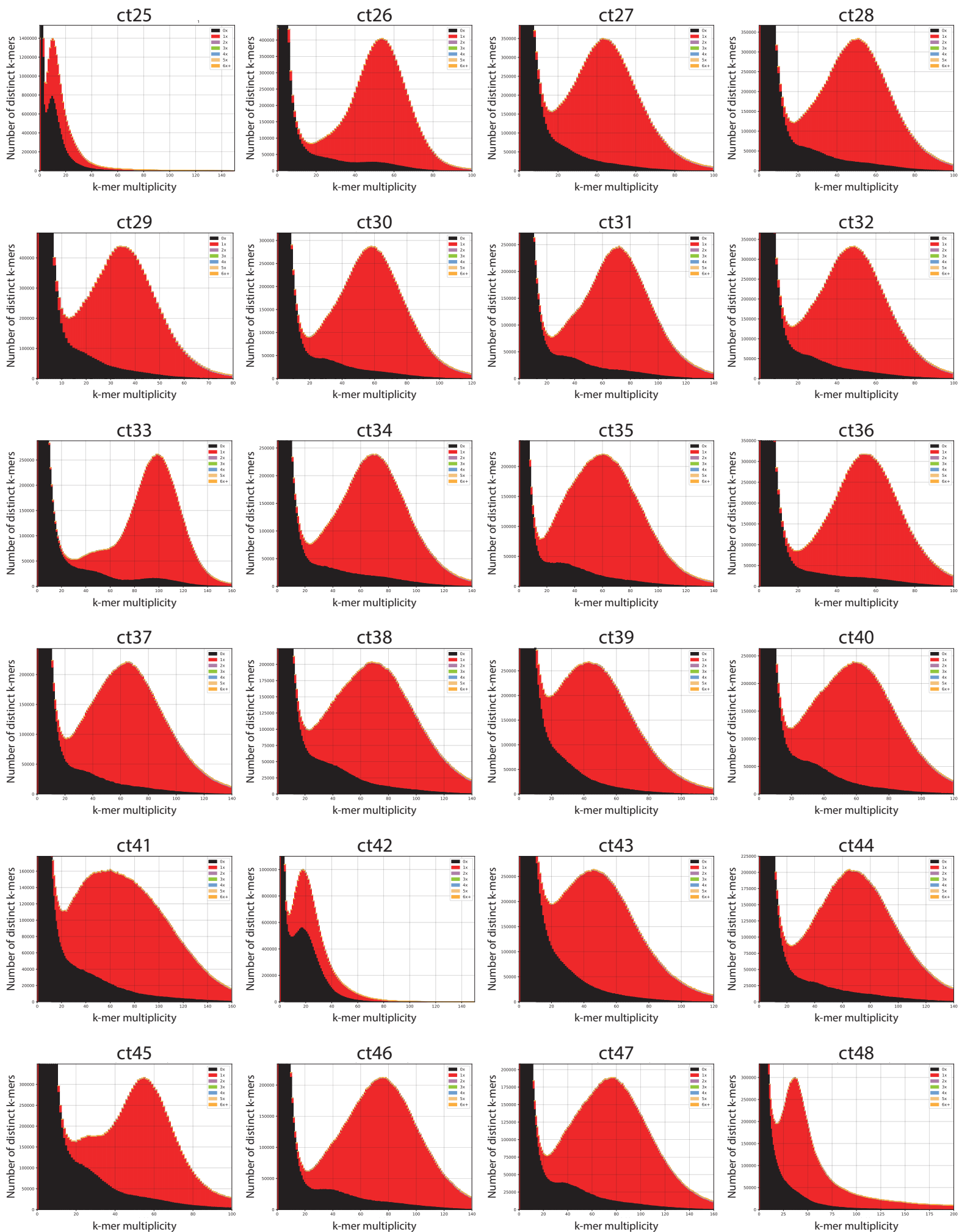

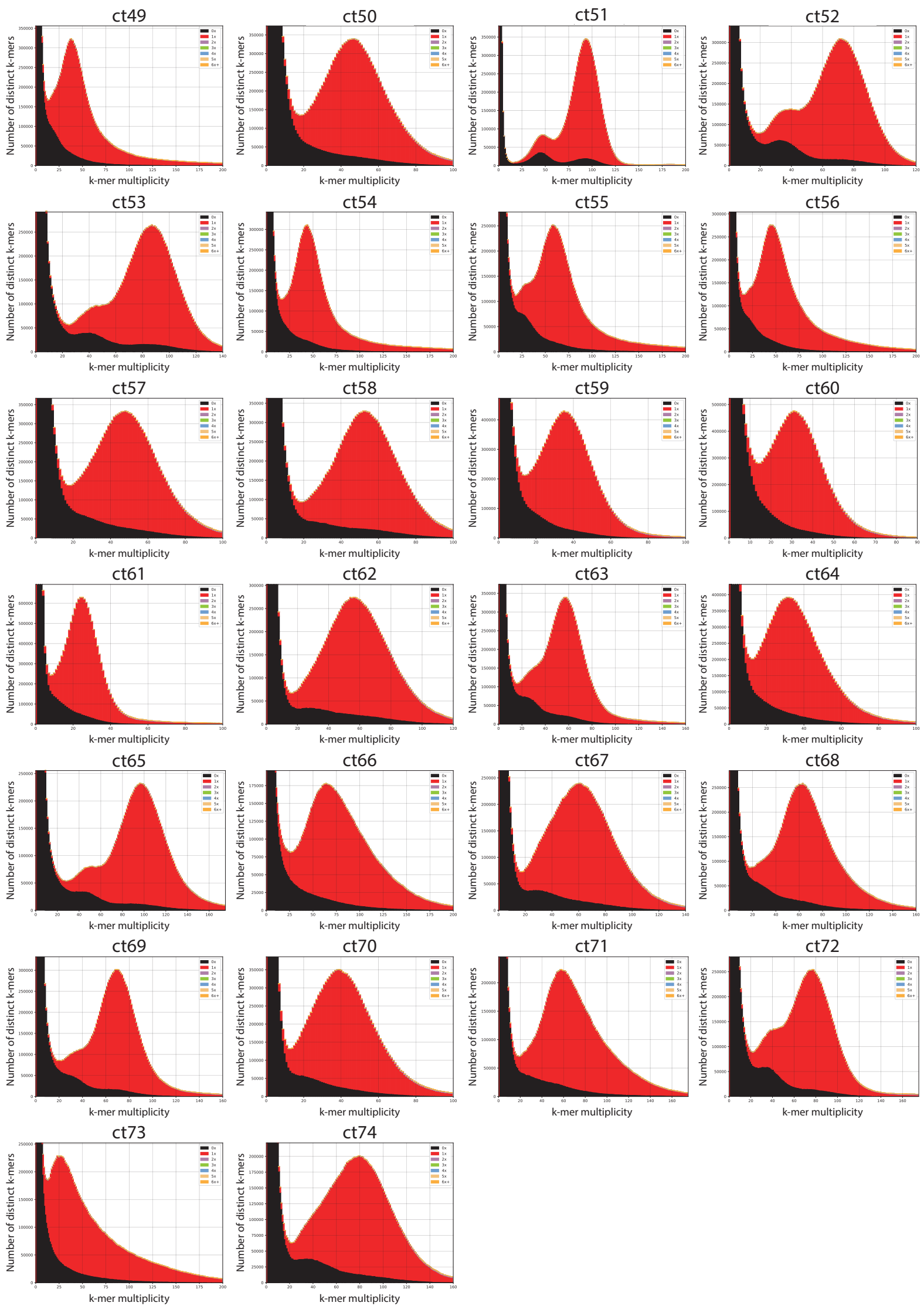

Supplement: S4 Fig — K-mer frequency distribution profiles are shown for all C. tropicalis isolates not in Fig 2. K-mer analysis was performed with the k-mer Analysis Toolkit (KAT [82]). For each isolate, the number of distinct k-mers of length 27 bases (27-mers) is displayed on the Y-axis and k-mer multiplicity (depth of coverage) is displayed on the X-axis. K-mers that are present in the reference genome with a frequency of 1 (i.e. 1X) are shown in red, and k-mers that are absent from the reference genome (i.e. 0X) are shown in black. In the non-hybrid (AA) isolates, there is no bimodal pattern observed, unlike in the hybrid isolates. (PDF) [file ppat.1009138.s005.pdf]

A

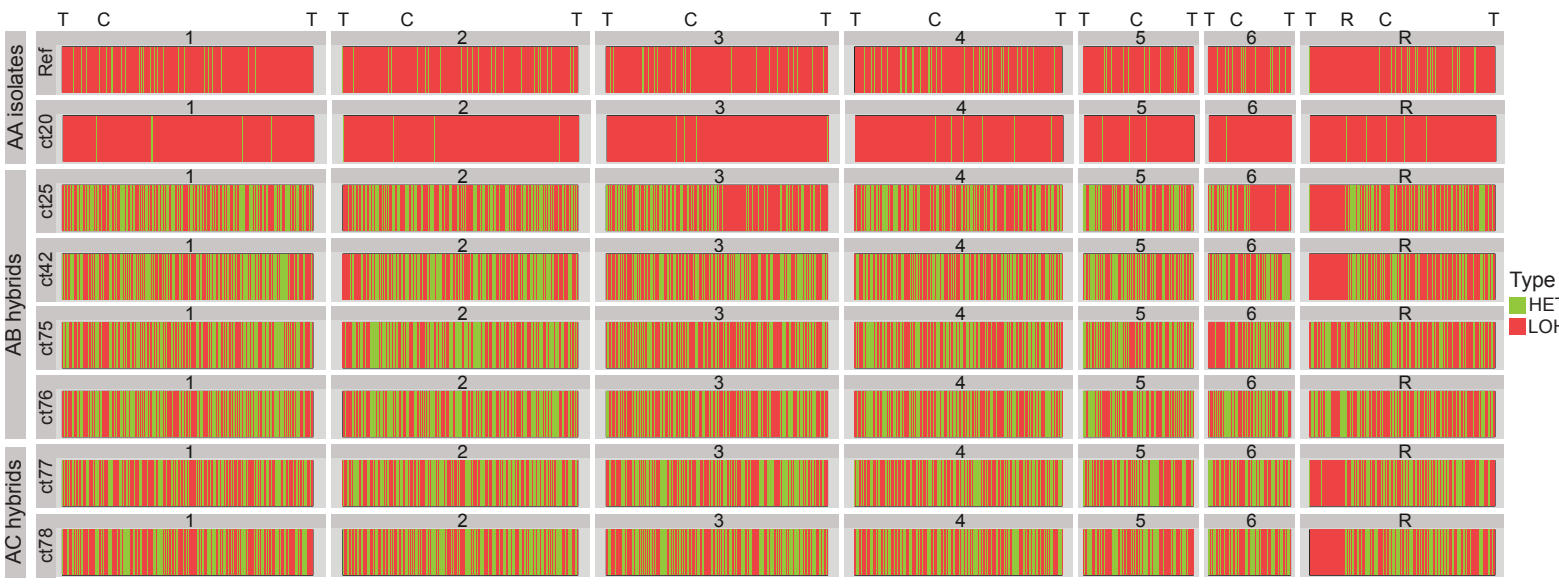

B

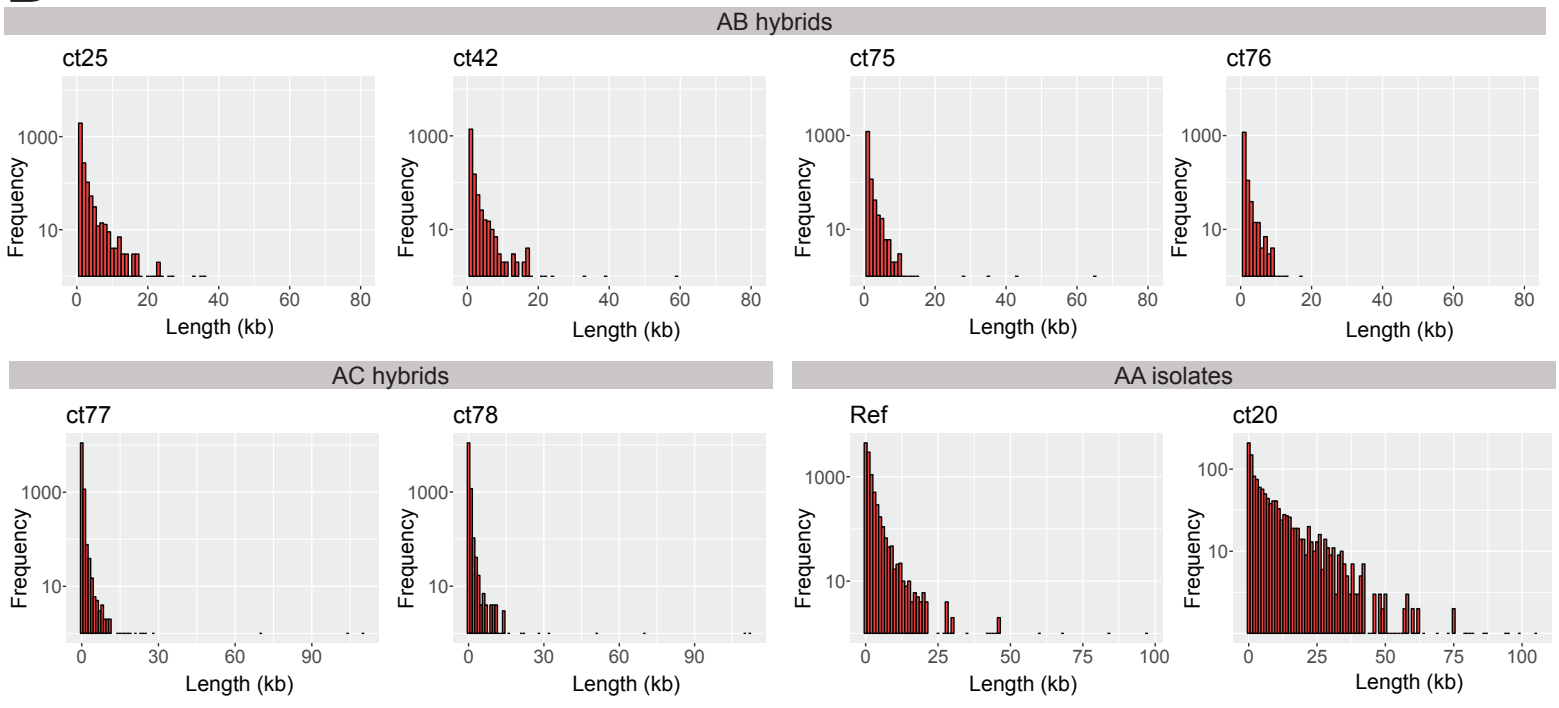

Supplement: S5 Fig — (A) The same patterns of LOH and heterozygosity are observed using an updated reference genome assembly. LOH was re-analyzed using an updated chromosome-level assembly from Guin et al [39]. The seven chromosomes in the reference genome are displayed horizontally from left to right and labelled from 1 to 6, plus chromosome R. Chromosomes in the alternative reference genome map to scaffolds in the original reference genome as follows; chr1:scaffold 2, chr2: scaffolds 5 and 6, chr3: scaffold 1, chr4: scaffold 3, chr5: scaffold 8, chr6: scaffold 7, chrR: scaffold 4. LOH blocks are shown in pink and heterozygous (“HET”) blocks are shown in green. Centromere positions are indicated with “C”, telomere positions are indicated with “T” and the rDNA locus is indicated with “R”. Isolates are labelled on the left-hand side. The re-sequenced reference strain C. tropicalis MYA-3404 (labelled as “Ref”) is shown as a representative of the non-hybrid (AA) isolates. The same patterns of LOH/heterozygosity are observed in the AA, AB and AC isolates when using the alternative reference as when using the original reference genome. (B) The length of LOH blocks are unchanged when analyzed using an updated reference genome assembly. The histograms show the frequency of LOH blocks of different lengths in the six hybrid isolates and two AA (non-hybrid) isolates, the re-sequenced reference strain C. tropicalis MYA-3404 (labelled as “Ref”) and C. tropicalis ct20. Frequency is shown on a log scale on the Y-axis while length in kilobases (kb) is shown on the X-axis, with a bin width of 1000 bp. The average length of LOH blocks in the hybrid isolates ranges from 289–417 bp, a difference of only a few base pairs from the analysis using the original reference genome. The same patterns are observed in the AA and hybrid isolates when using the updated reference genome. (PDF) [file ppat.1009138.s006.pdf]

A

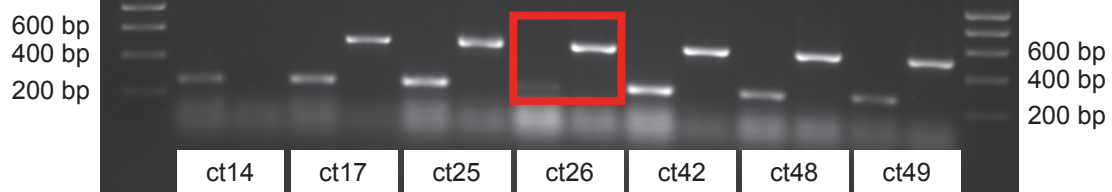

B

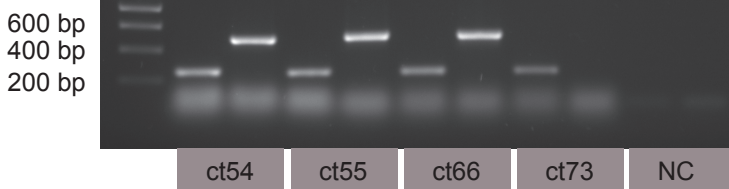

Supplement: S6 Fig — The gel shows the results of the colony PCR amplification of the MTL in eleven C. tropicalis isolates (labelled in grey or white boxes). Hyperladder is shown on the left- and right-most column of the gel on both rows, with the sizes of the bottom three markers (200 bp, 400 bp and 600 bp) marked. Two reactions were performed for each isolate—one using primer pairs MTLa1F and MTLa1R to amplify the MTLa1 gene (lane marked “a”) and MTLα2F and MTLα2R to amplify the MTLα2 gene (lane marked “α”), as described in Xie et al. [21]. A band of 253 bp is expected in the “a” lane for isolates with at least one copy of the MTLa1 gene and a band of 525 bp is expected in the “α” lane for isolates with at least one copy of the MTLα2 gene. Negative control (all components of PCR mix excluding input DNA) is marked as “NC” on the bottom row, with one lane for each primer set (marked “a” and “α”). Most isolates are heterozygous, but C. tropicalis ct14 and ct73 are homozygous for MTLa. The octoploid isolate C. tropicalis ct26 has a strong positive signal for MTLα (lane marked “α”) and a weak positive signal for MTLa (lane marked “a”), highlighted with a red box. The genome assembly contains one full copy of OBPa, and partial copies of the remainder of the MTLa genes (PAPa, PIKa, MTLa2 and MTLa1). The five MTLa genes are scattered across five low-coverage contigs (coverage 1.3X - 2X), most of which are only the length of the gene itself. One gene, MTLa2, is split across two scaffolds. It is possible that there is one copy of MTLa and up to seven copies of MTLα, resulting in low sequencing coverage of the MTLa locus. (PDF) [file ppat.1009138.s007.pdf]

A

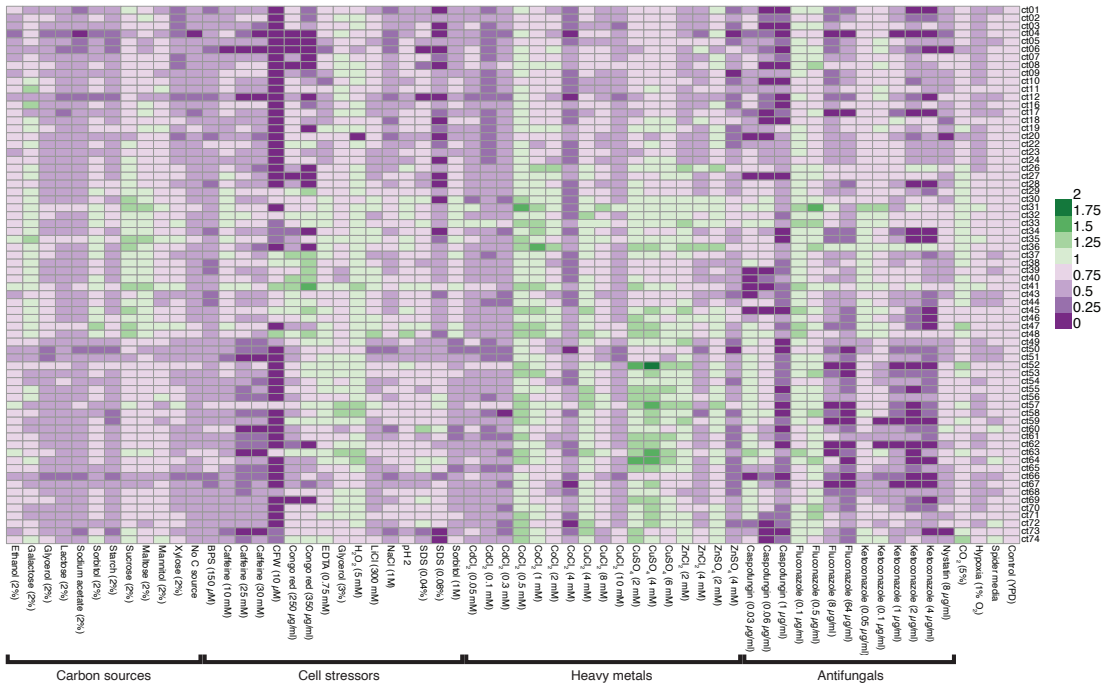

B

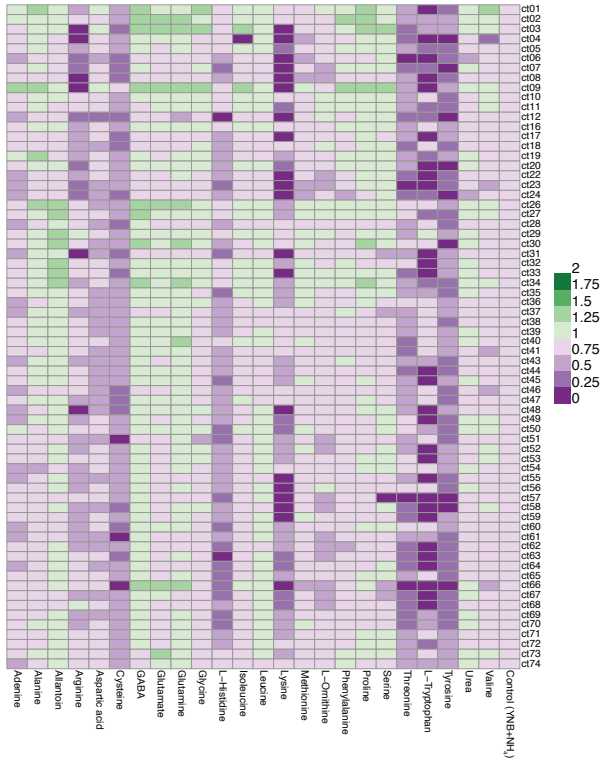

Supplement: S7 Fig — 68 C. tropicalis isolates were grown on YPD (A) or YNB with ammonium (NH4) (B) solid agar media as a control, and compared to strains growing on solid agar media containing different stressors. Pictures were taken after 48 hours and colony size and growth scores were measured using SGAtools [80]. Heatmaps show the normalized raw colony size in various tested growth conditions. Isolates are represented in rows, and are ordered alphabetically by strain alias. Growth conditions are shown in columns. Increased growth relative to YPD or YNB + NH4 is shown in green (1–2) and decreased growth is shown in purple (0–1). Major differences are observed between isolates growing in the presence of cell wall stressors (calcofluor white, congo red, sodium dodecyl sulphate, caffeine), and antifungal drugs (ketoconazole, caspofungin, fluconazole). Hybrid isolates and engineered lab isolates were excluded from this analysis. (PDF) [file ppat.1009138.s008.pdf]

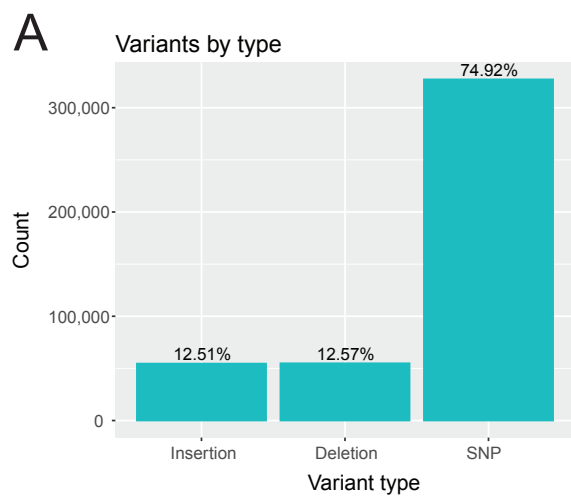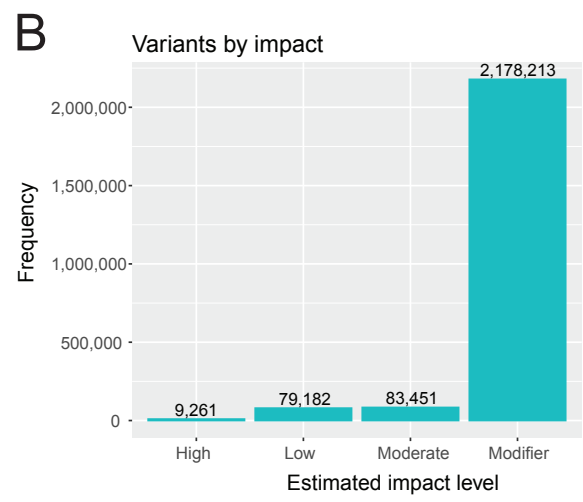

Supplement: S8 Fig — (A) The majority of variants in non-hybrid (AA) C. tropicalis isolates are single nucleotide polymorphisms (SNPs). Variants were called in all non-hybrid isolates using the Genome Analysis Toolkit [42] and annotated with SnpEff [46]. Variant type is shown as a barplot, with variant categories on the X-axis and variant count on the Y-axis. Approximately 75% of all annotated variants are SNPs, 12.51% are insertions and 12.57% are deletions. (B) Identification of high-impact variants. 9,261 high-impact variants were identified across 68 non-hybrid C. tropicalis isolates. Variant classification according to SnpEff is shown as a barplot, with estimated impact level categories on the X-axis and variant count on the Y-axis. Precise counts are shown above each bar. 9,261 variants were annotated as “high impact.” These variants are predicted to have a major impact on protein function (e.g. gain or loss of start or stop codon, frameshifts, or splice site variants). These variants were analyzed for potential genotype-phenotype correlations. (PDF) [file ppat.1009138.s009.pdf]
